# Five-year survival and associated factors in women treated for cervical cancer at a reference hospital in the Brazilian Amazon

**DOI:** 10.1371/journal.pone.0187579

**Published:** 2017-11-16

**Authors:** Saul Rassy Carneiro, Marcela de Araújo Fagundes, Pricila de Jesus Oliveira do Rosário, Laura Maria Tomazi. Neves, Givago da Silva Souza, Maria da Conceição Nascimento Pinheiro

**Affiliations:** 1 Hospital Universitário João de Barros Barreto, Universidade Federal do Pará, Belém, Pará, Brasil; 2 Núcleo de Medicina Tropical, Universidade Federal do Pará, Belém, Pará, Brasil; 3 Faculdade de Fisioterapia e Terapia Ocupacional, Universidade Federal do Pará, Belém, Pará, Brasil; Fondazione IRCCS Istituto Nazionale dei Tumori, ITALY

## Abstract

Cervical cancer (CC) is the most common type of cancer in women and is the third leading cause of death in most developing countries, causing more than 288,000 deaths in women worldwide each year. The most favourable survival rate is in developed countries, since CC mortality has recently declined in those countries. The purpose of this study was to determine the survival rate and associated factors of CC patients at a reference hospital in the Amazon region. The patient sample included records of 339 patients with cervical cancer who had been hospitalized in Belém, Pará, Brazil from January 2005 to December 2010; the socioeconomic and clinical data were collected between June and September 2016. A survival rate of approximately 84% was observed, and it was found that disease stage (p <0.01), metastasis (p <0.01) and readmission (p <0.01) had significant influences on patient outcome. The impact of these factors on the general survival rate was higher in the Amazon region compared with other regions of Brazil, and the primary survival factors were associated with earlier stages of the disease. However, more national studies are needed on this subject. Our findings may contribute to the development of regional strategies for the prevention of cervical cancer, a reduction in its incidence and mortality rate, an increase in survival time and an improvement in the quality of life of these women.

## Introduction

Oncological diseases are a leading cause of death worldwide. In 2012, approximately 8.2 million people died of cancer, and this situation will become more alarming over the next two decades based on estimates of 22 million new cases and 13 million deaths per year [1]. This increase is due to both the growth and ageing of the population, particularly in less developed countries, as well as to an increase in the practice of lifestyles known to intensify the risk of cancer, such as smoking, poor diet, sedentarism and reproductive changes that include fewer children and later age at first birth [2].

Cervical cancer (CC) is the most common type of cancer in women and is the third leading cause of death in most developing countries. Approximately 500,000 new cases are diagnosed each year, 80% of which are in less developed regions. These cases result in more than 288,000 deaths each year worldwide, the majority of which occur in the poorest regions: South Asia, Sub-Saharan Africa and parts of Latin America [1,3]. In Brazil, cervical cancer is the fourth most frequent type of cancer in women, behind only breast, intestinal and lung cancer; in 2016, 16,340 new cases of CC occurred in Brazil, which accounted for 7.9% of all CC cases. Aside from non-melanoma skin cancer, cervical cancer is the most frequently diagnosed cancer type in the Brazilian Amazon (23.97/100,000 women). Pará is the second largest state in both Brazil and the Amazon region; it also has the fourth highest number of CC cases, with approximately 820 new cases in 2016 (20.52/100,000) [4]. Recent years have seen a reduction in worldwide CC mortality rates; this reduction may be associated with improved socioeconomic conditions and/or better screening, which is due in large part to the widespread use of Pap smears for the prevention and early detection of CC [5,6].

The five-year survival analysis is considered a standard indicator for the comparison of cancer survival rates in population studies where the cause of death is unknown or unreliable. The most favourable survival rates can be found in developed countries, while the worst are found in certain developing countries. A number of different factors may be associated with the overall survival of patients with cervical cancer [2,3,6,7]. Thus, the present study estimated the five-year survival rate and the associated factors in patients diagnosed with CC at a reference hospital in the Amazon region.

## Materials and methods

### Study design

This study was conducted according to the Research Guidelines Involving Human Beings (Resolution CNS 466/2012) of the National Health Council in Brazil, and commenced only after approval from the Research Ethics Committee of the João de Barros Barreto University Hospital, in Belém, State of Pará, under opinion 1,472,289.

The objective of this retrospective cohort study of a hospital series [8] was to analyse the survival of CC patients treated at Ophir Loyola Hospital, which is a CC treatment referral hospital in the Amazon region. The cases were selected through an active search of records in the Medical Statistics Analysis Department of women hospitalized between January 2005 and December 2010, patient records were kept anonymous and their identifications were made through numerical records. Data were collected between June and September 2016 and stored in a file prepared by the authors.

The study variables were as follows: age (18–29 years, 30–50 years and over 50 years), marital status (single, married, divorced, widowed), occupation (employed, housewife/unemployed, retired), origin (Greater Belém, interior of Pará), number of children (no children, one child, more than one child), histological type (Squamous cell carcinoma, adenocarcinoma, other types), tumour stage, which was divided into 2 groups according to the International Federation of Gynecology and Obstetrics (FIGO) 2009 staging model (Group 1—from IA to IB, minor lesions with a lower degree of tissue infiltration, still restricted to the cervix, and Group 2—from IIA to IVB, lesions with a higher degree of infiltration and extending beyond the cervix) [9,10], treatment (exclusive, combined, none), survival time, smoking (nonsmoker, smoker/ex-smoker), comorbidities or associated chronic non-communicable diseases and infectious diseases (none, one, two or more), metastasis (yes or no) and readmission (yes or no). Patients whose medical records contained incomplete data and or incomplete five-year follow-up information were excluded.

### Statistical analysis

The randomization of hospital records was performed through the probabilistic sampling process with replacement, and a test for simple random samples was used; a sample error of 5% and a significance level of 95% were used [11]. The sample consisted of CC patients who were admitted between 2005 and 2010 and whose medical records included histopathological or anatomopathological examinations. Incomplete records or cases in which treatment was abandoned before the completion of the five-year follow-up time were excluded.

The time of survival was calculated as the interval between the date of diagnosis (by biopsy or surgery) in the hospital record and the date of death or the end of follow-up. The maximum follow-up time was five years; the case of any patient who remained alive after this point was closed.

The data were stored in an Excel^®^ 2013 spreadsheet (Microsoft Corporation, CA, USA). Case selection was randomized, and if a selected medical record was excluded, the immediate subsequent case was analysed. SPSS version 22.0 (International Business Machines, NY, USA) was used for data analysis and for the production of graphics. The log-rank test was used to analyse each independent variable along with the survival time; variables with a significance level below 20% were included in a Cox Regression analysis, which estimated the proportionality of the risks over the observation period [11]. Cox model variables with a p-value <0.05 were considered to have actually interfered with the survival of the patients.

## Results

The data search returned a total of 1851 CC cases that presented between 2005 and 2010, of which 339 were included in this study. During the five-year follow-up period of this cohort, 88 deaths occurred. We compared the survival function of the subjects using a Kaplan-Meier plot. Patient survival was analysed according to each variable to determine whether a specific variable significantly interfered with the survival of the subjects.

The characteristics of the included patients are presented in Table 1. The mean age of the sample was 49.67 (± 13.89) years. The origin of the patients was classified as either Greater Belém (n = 159, 46.9%) or the interior of Pará (n = 180, 53.1%). Most patients were married (n = 148, 43.6%) or single (n = 143, 42.2%), with 12.1% (n = 41) widowed and 2.1% (n = 7) divorced. According to occupation, 64.0% were housewives or unemployed (n = 217), 111 were employed (32.7%) and 3.3% (n = 11) were retired.

**Table 1:** Sample characteristics.

Characteristic	n (%)	Characteristic	n (%)
**Origin**		**Comorbidities**	
*Belém (Capital of Pará State)*	159 (46.9)	*None*	212 (62.5)
*Small Cities within Pará*	180 (53.1)	*One*	96 (28.4)
**Marital status**		*Two or more*	31 (9.1)
*Single*	143 (42.2)	**Smoking**	
*Married*	148 (43.6)	*Smoker/Ex-smoker*	178 (52.5)
*Divorced*	7 (2.1)	*Nonsmoker*	161 (47.5)
*Widowed*	41 (12.1)	**Children**	
**Occupation**		*No children*	26 (7.7)
*Housewife/Unemployed*	217 (64.0)	*One child*	18 (5.3)
*Employed*	111 (32.7)	*More than one child*	295 (87.0)
*Retired*	11 (3.3)	**Treatment**	
**Histological Type**		*None*	15 (4.5)
*Squamous cell carcinoma*	318 (93.8)	*Only 1 treatment*	169 (49.8)
*Adenocarcinoma*	19 (5.6)	*More than 1 treatment*	155 (45.7)
*Other*	2 (0.6)	**Readmission**	
**Staging**		*Yes*	71 (20.9)
*Group 1*	235 (69.3)	*No*	268 (79.1)
*Group 2*	104 (30.7)		
**Metastasis**		**Total**	339
*Yes*	63 (18.6)		
*No*	276 (81.4)		

The histological types of cervical cancer were classified as: squamous cell carcinoma (n = 318, 93.8%), adenocarcinoma (n = 19, 5.6%) or other types (n = 2, 0.6%). Regarding disease stage, 69.3% of the sample (n = 235) was classified as Group 1, and 30.7% (n = 104) was classified as Group 2. No metastasis was observed in the majority of patients, 81.4% (n = 276) with metastasis vs. 18.6% without (n = 63). Patient comorbidities included diabetes, hypertension, and HPV infection, among others. Statistical analysis involved the separation of patients with no comorbidities (n = 212, 62.5%) from patients with only one comorbidity (n = 96, 28.4%) and from patients with two or more associated comorbidities (n = 31, 9.1%).

The mean number of children per patient was 4.98 (± 3.5); 7.7% (n = 26) were nulliparous, 5.3% (n = 18) were primiparous and 87.0% (n = 295) were multiparous. Smokers and ex-smokers accounted for 52.5% (178) of the sample compared with non-smokers, who accounted for 47.5% (n = 161). The treatments for CC performed at this hospital included: surgery, chemotherapy, radiotherapy and brachytherapy. Treatment choice varied greatly and depended primarily on the stage of the disease. It was observed that some patients received only one treatment type (n = 169, 49.85%), while others received a combination of two or more treatments (n = 155, 45.72%); a small percentage (n = 15, 4.43%) received none of these treatment types, which may have been due to the presence of advanced, incurable disease. In all, 71 patients (20.9%) were readmitted due to cancer-related complications.

Table 2 presents the results of the survival analysis, including the analysis of the following subgroups: number of cases, deaths, mean survival, log-rank test (for comparisons of the risk function estimates for each observed event) [5] and the Cox regression analysis, which determined the independent variables that acted as intensifiers during the time between diagnosis and death. The statistical significance was set at p <0.05 (95% CI). The log-rank test revealed that the variables of marital status (p <0.001), histological type (p <0.05), stage (p <0.001), metastasis (p <0.001), treatment type (p <0.001) and readmission (p <0.001) influenced the risk of death in these patients. Based on these results, we analysed the influence of each independent variable on survival time, and found that disease stage (p <0.001), metastasis (p <0.01) and readmission (p <0.01) significantly impacted patient survival.

**Table 2:** Bivariate analysis of survival and risk ratio in women with cervical cancer, plus cox regression for the multivariate analysis. Belém, Pará, Brazil, 2005–2010.

Variable	n cases (%)	n deaths (%)	SV rate (%)	Mean SV (months)	Log- rank test (p)	Cox Regression	
**Age range (years)**					0.415	-	
*21–30*	28 (8.3)	10 (35.7)	64.3	44			
*30–50*	159 (46.9)	38 (23.9)	76.1	49			
*50–00*	152 (44.8)	40 (26.3)	73.7	47			
**Origin**					0.272	-	
*Belém* *(Capital of Pará State)*	159 (46.9)	46 (28.9)	71.1	47			
*Small Cities within Pará*	180 (53.1)	42 (23.3)	76.7	49			
**Marital Status**					**0.001**	0.973	
*Single*	143 (42.2)	42 (29.4)	70.6	48			
*Married*	148 (43.7)	28 (18.9)	81.1	50			
*Divorced*	07 (2.1)	05 (71.4)	28.6	23			
*Widowed*	41 (12.1)	13 (31.7)	68.3	44			
**Occupation**					0.259	-	
*Employed*	111 (32.7)	34 (30.6)	69.4	45			
Housewife/ *Unemployed*	217 (64.0)	50 (23.0)	77	49			
*Retired*	11 (3.2)	4 (36.4)	63.6	49			
**Histological Type**					**0.018**	0.768	
*Squamous cell carcinoma*	318 (93.8)	78 (24.5)	75.5	48			
*Adenocarcinoma*	19 (5.6)	10 (52.6)	47.4	37			
*Other*	2 (0.6)	0 (0)	100	60			
**Staging**					**0.000**	**0.000**	
*Group 1*	235 (69.3)	18 (7.7)	92.3	57			
*Group 2*	104 (30.7)	70 (67.3)	32.7	22			
**Metastasis**					**0.000**	**0.009**	
*No*	276 (81.4)	51 (18.5)	81.5	52			
*Yes*	63 (18.6)	37 (58.7)	41.3	30			
**Comorbidities**					0.605	-	
*None*	212 (62.5)	54 (25.5)	74.5	48			
*One*	96 (28.3)	24 (25)	75	49			
*Two or more*	31 (9.2)	10 (32.3)	67.7	43			
**Children**					0.238	-	
*No children*	26 (7.7)	6 (23.1)	76.9	49			
*One child*	18 (5.3)	8 (44.4)	55.6	42			
*More than one child*	295 (87.0)	74 (25.1)	74.9	48			
**Smoking**					0.201	0.806	
*Never-smoker*	161 (47.5)	35 (21.7)	78.3	49			
*Smoker/Ex-smoker*	178 (52.5)	53 (29.8)	70.2	46			
**Treatment**					**0.000**	0.250	
*Exclusive*	169 (49.9)	26 (15.4)	84.6	52			
*Combined*	155 (45.7)	48 (31.0)	69	48			
*None*	15 (4.4)	14 (93.3)	6.7	5			
**Readmission**					**0.000**	**0.007**	
*No*	268 (79.1)	52 (19.4)	80.6	50			
*Yes*	71 (20.9)	36 (50.7)	49.3	39			
**Total**	**339 (100)**	**88 (26)**	74	**48**	**-**		

SV rate = Survival rate

**Fig 1:**
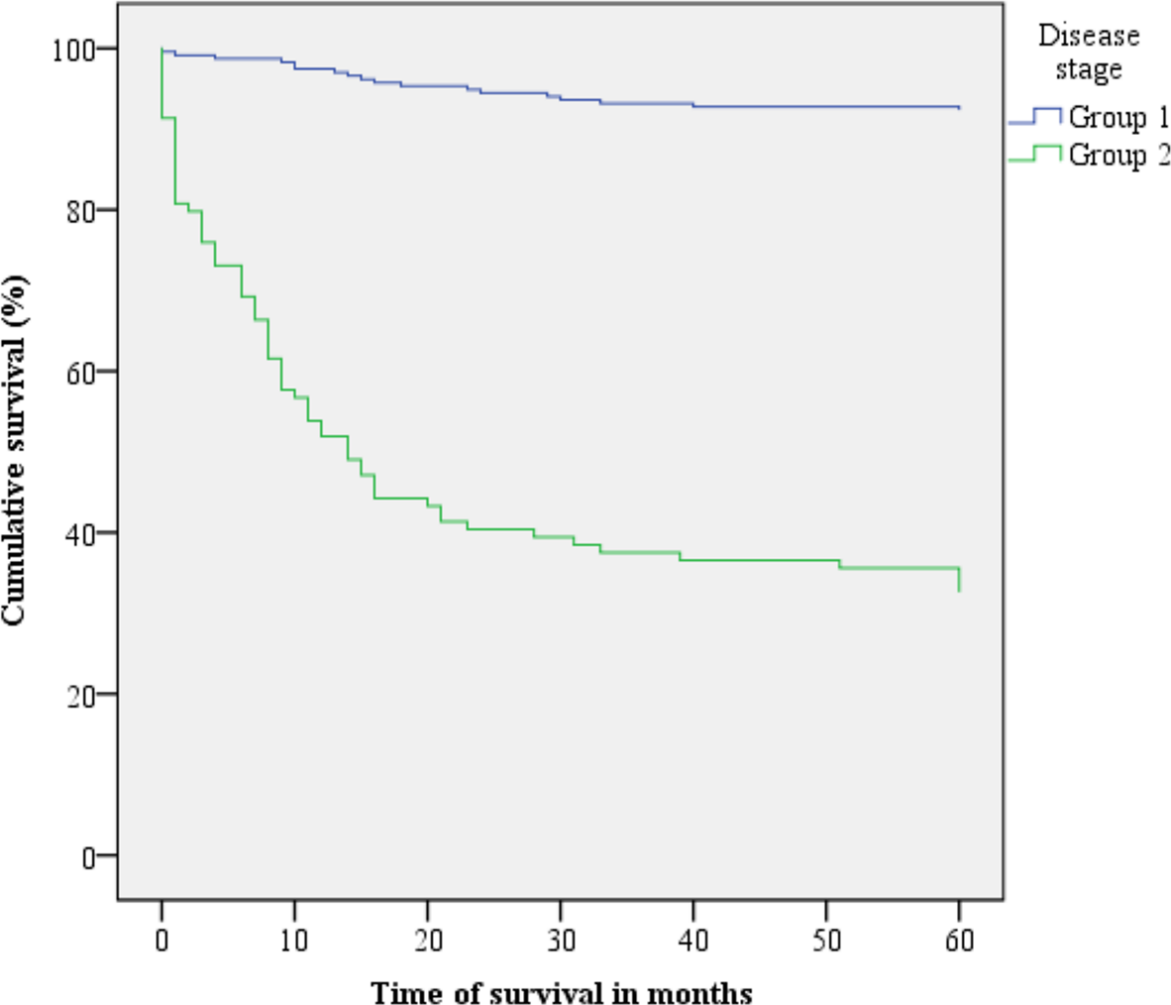
Kaplan-Meier survival curves for cervical cancer patients according to disease stage. Belém, Pará, Brazil. 2005–2010. Group 1: stages IA to IB. Group 2: stages IIA to IV B.

**Fig 2:**
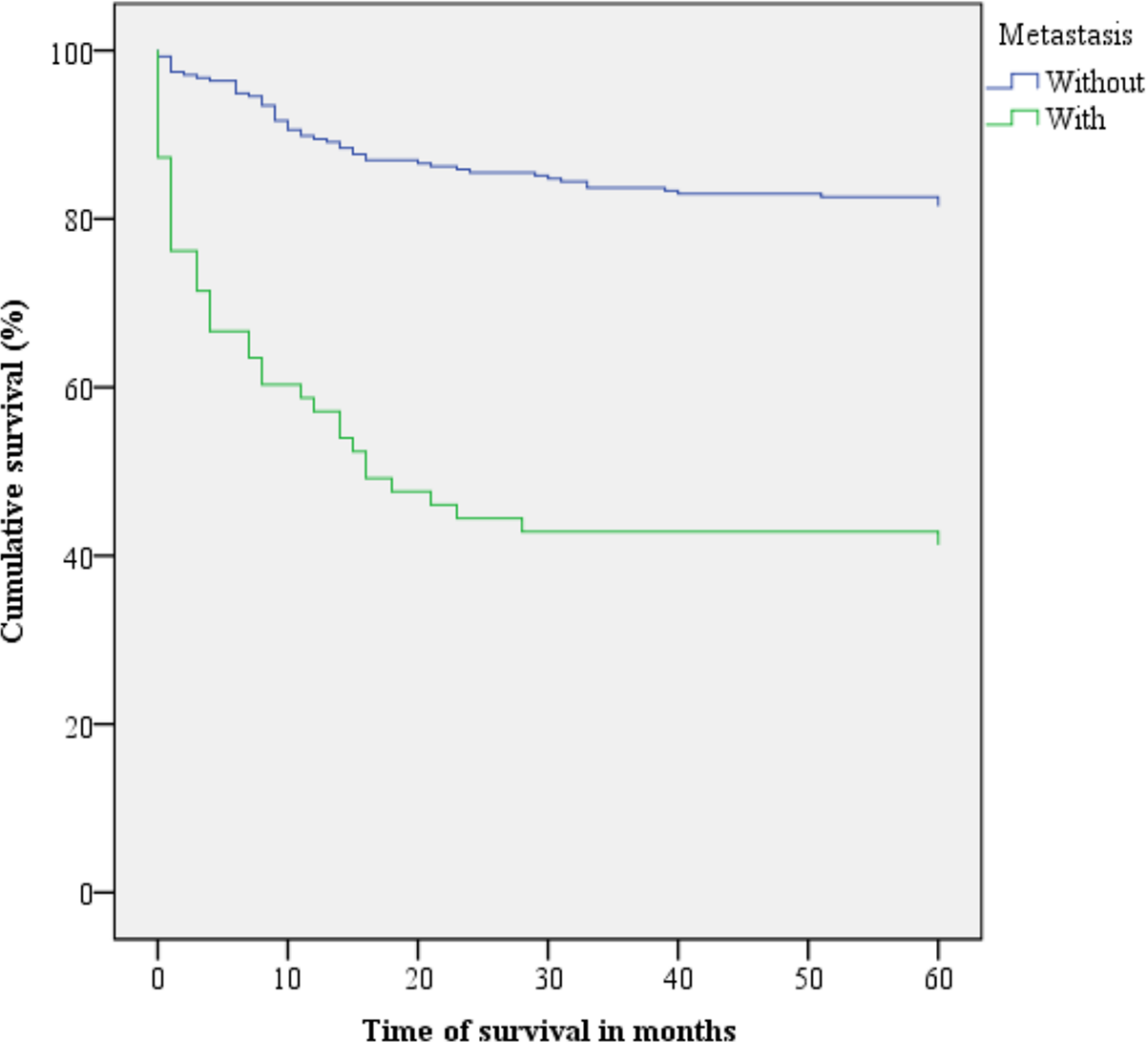
Kaplan-Meier survival curves for cervical cancer patients with and without metastasis. Belém, Pará, Brazil. 2005–2010.

**Fig 3:**
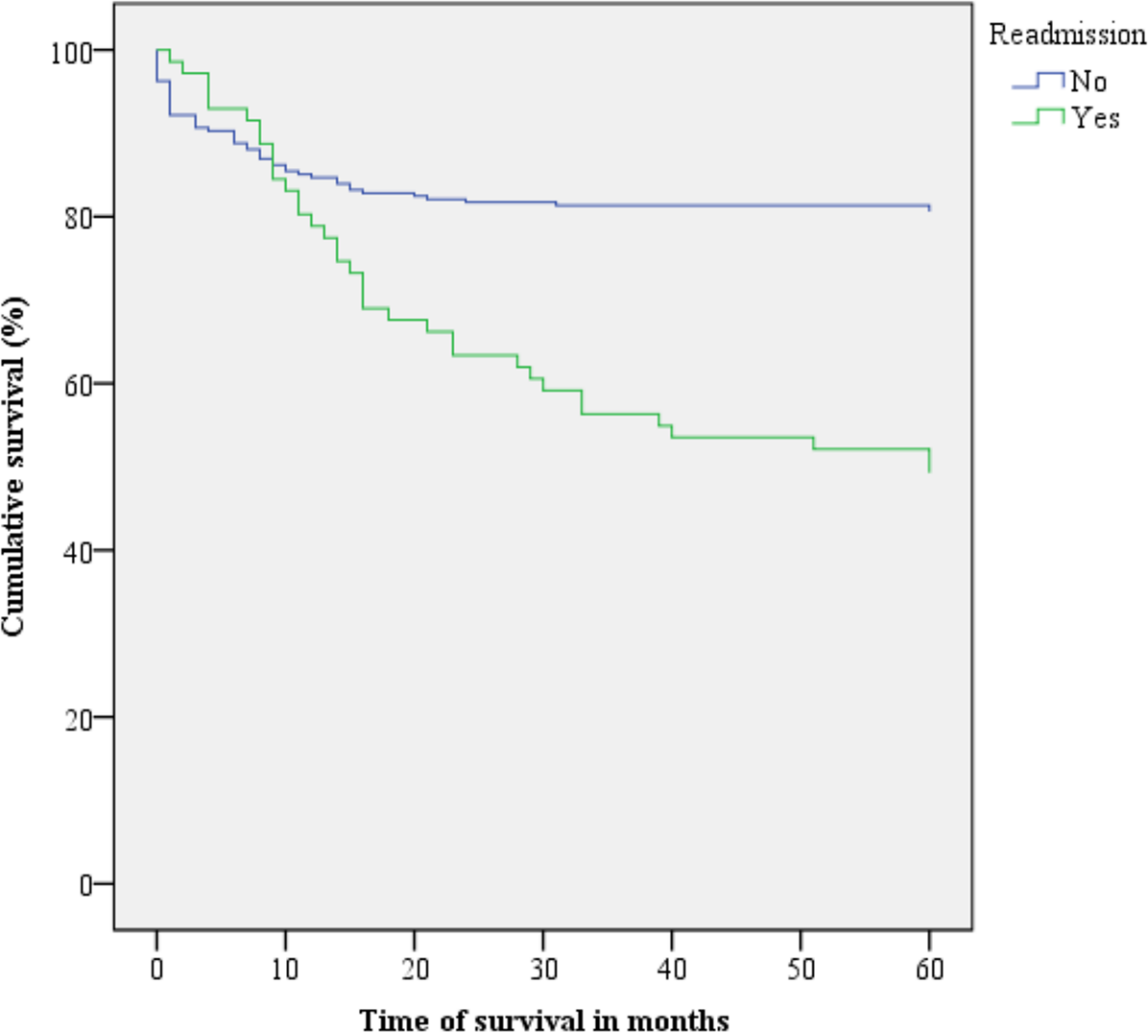
Kaplan-Meier survival curves for cervical cancer patients according to readmission. Belém, Pará, Brazil. 2005–2010.

**Fig 4:**
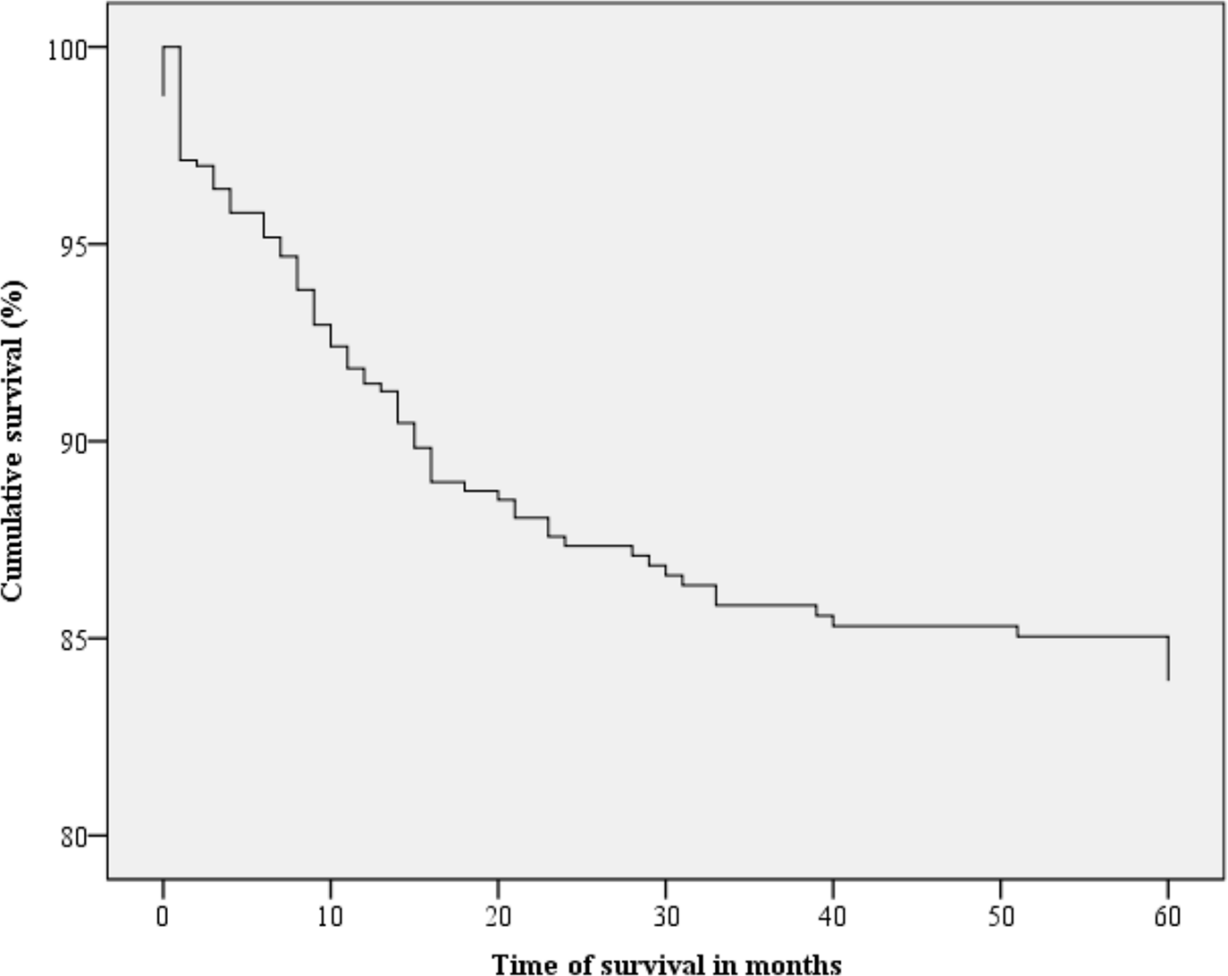
Kaplan-Meier survival curves for cervical cancer patients according to the mean of the covariates. Belém, Pará, Brazil. 2005–2010.

Figs 1–3 present the Kaplan-Meier curve with five-year survival estimates for the following variables: stage (Group 1 or 2), metastasis and readmission. Fig 4 presents the overall survival function during the study period with respect to the independent variables.

## Discussion

Survival studies provide essential information about factors that may be associated with disease prevention and treatment, which can lead to more effective health policies and to health systems with better patient survival rates [12]. A high survival rate was observed in this study, and the factors associated with this survival were disease stage, metastasis and readmission.

The 84% survival rate in our sample may be considered high compared with the survival rates in developed countries and in other regions of Brazil. According to data from the US National Center for Health Statistics, the relative five-year survival rate of CC patients is 68% [13].

A study conducted in northeast Brazil that involved 1515 CC cases (2003–2008) found a 62.21% five-year survival rate in those who underwent treatment [14]. Carmo and Luiz analysed 3341 CC cases in the state of Rio de Janeiro (1999–2004) and found a five-year survival rate of 48% [15]. A survey of more than 25 million cancer cases recorded in the national information systems of 67 countries between 2005 and 2009 revealed that CC survival rates in developed countries such as the United States (62.8%), Japan (66.3%) and Australia (67.1%) were higher than those of developing countries such as South Africa (54.9%), Chile (50.9%) and Brazil (61.1%) [12].

Disease staging, which takes into account tumour size, lymph node involvement and the presence of distant metastases, has been shown to be an important prognostic factor in CC patient survival. The survival rates of Group 2 (stages IIA to IVB) and Group 1 (IA to IB) patients were 32.7% and 92.3%, respectively. A 2009 study in California, which included 353 CC survivors, also indicated that cancer stage is a significant survival factor [10,16]. In Nakagawa’s cohort sample of 55 patients, the five-year survival rate for later-stage CC was 61.9%, whereas for earlier stages, the five-year survival rate was 100%. These data demonstrate that early diagnosis reduces the risk of death and that when the disease is treated in the initial phase, it can be completely cured [17].

The survival rate of patients with metastasis was unfavourable, but the survival rate of metastasis-free patients was 81.5%, i.e., the majority of deaths occurred in patients with later-stage CC. This corresponds with the results of the study by Ashing-Giwa et al., who found a five-year survival rate of over 90% for early-stage CC without metastasis [16]. Lorin et al. studied 1019 cases of invasive cervical cancer between 1998 and 2010, and found that after surgery, stage was the most important prognostic factor, as only 15% of patients with metastasis survived up to 60 months [18].

Readmission was also demonstrated to be an important survival factor for the present sample. It was observed that when CC patients were readmitted to the hospital at least once after discharge, they had a lower survival rate than those who were not readmitted. Lipsitz et al. found that cancer patients who were readmitted within 30 days after discharge belong to a higher risk group [19]. According to Donzé, Lipsitz and Schnipper, the readmission of cancer patients may occur for several reasons, including malignancy, complications due to cancer-associated comorbidities and complications due to the sometimes aggressive treatments that they undergo. Sometimes these patients are readmitted to receive treatments such as chemotherapy, and other times, they are readmitted for end-of-life palliative care [20].

Socioeconomic, demographic and medical variables are known to be associated with survival [21,22]. However, in this study, factors such as age, marital status, origin, occupation, histological type and smoking status showed no influence on survival. It was observed that retired and divorced women had lower survival rates than other women. One possible cause for this might be that social support is a critical factor during a treatment series [9]. Considering age as a factor, the highest risk of death was found between those who were 30–50 years of age, which may reflect the higher number of cases in this age group [17,18,23]. In addition, Bifulco et al. observed a significant worsening of the quality of life of women at younger ages with less adjustment to well-being levels after diagnosis and treatment of cancer than middle-aged patients [24]. National and international guidelines recommend Pap smear screening and appropriate care for women with possible precursor or invasive lesions beginning at age 25 and 21, respectively, this care is important for early diagnosis and may increase the success of treatment and improve the survival rates of these patients [25].

Although certain associated diseases and smoking are known to be important factors that contribute to an increased risk of cancer, our sample suggests that the presence of these diseases is secondary to the abovementioned clinical factors. Ibfelt et al. found that socioeconomic differences are more pronounced in different stages of cancer, as are to a lesser degree, smoking status and comorbidities [21]. Furthermore, histological type demonstrated no important association with patient survival. Squamous cell carcinoma was the most frequent type observed in the sample, and the survival rate of patients with this subtype was lower than that of patients with adenocarcinoma. A survey in Istanbul found that 86.9% of the patients were diagnosed with squamous cell carcinoma, while 13.1% were diagnosed with adenocarcinoma, with overall five-year survival rates of 73% and 77%, respectively [26]. Rudtanasudjatum et al. surveyed 499 CC patients in Thailand and found no significant survival differences between those with squamous cell carcinoma and those with adenocarcinoma or other histological subtypes [27]. It is understood that these factors may influence analyses related to disease incidence and treatment optimization.

## Conclusion

The overall survival rate of CC patients was high compared with the survival rates in other regions of Brazil and in other developing countries. Factors relevant to patient survival were mainly related to clinical issues such as disease stage, metastasis and readmission. Other national studies should be conducted to identify not only the survival time, but the factors that influence it, which could contribute to regional strategies to combat CC, to reduce both the incidence and mortality rates, to increase the survival time and to improve the quality of life of these women.

## Limitations

This study was based on secondary data, which may have interfered with the observed results. We can cite as other limitations the lack of standardization in the completion of the included medical records, as well as in manual data recording and the responsible department’s storage system, which may have contributed to sample loss due to illegible data, although this did not exceed 15% of the total. The medical record data were collected and transcribed by different individuals, which could have resulted in errors despite their training.

## Supporting information

S1 File Database of the study. (XLSX)
